# Cardiovascular disease in patients with rheumatoid arthritis: results from the QUEST-RA study

**DOI:** 10.1186/ar2383

**Published:** 2008-03-06

**Authors:** Antonio Naranjo, Tuulikki Sokka, Miguel A Descalzo, Jaime Calvo-Alén, Kim Hørslev-Petersen, Reijo K Luukkainen, Bernard Combe, Gerd R Burmester, Joe Devlin, Gianfranco Ferraccioli, Alessia Morelli, Monique Hoekstra, Maria Majdan, Stefan Sadkiewicz, Miguel Belmonte, Ann-Carin Holmqvist, Ernest Choy, Recep Tunc, Aleksander Dimic, Martin Bergman, Sergio Toloza, Theodore Pincus, the QUEST-RA Group

**Affiliations:** 1 Hospital de Gran Canaria Dr. Negrin, University of Las Palmas de Gran Canaria, Barranco de la Ballena s/n 35011, Spain; 2 Jyväskylä Central Hospital, Jyväskylä, and Medcare Oy, Äänekoski, Finland; Jyväskylä Central Hospital, Keskussairaalantie 19, 40620 Jyväskylä, Finland; 3 Research Unit, Spanish Foundation of Rheumatology, C/Marqués de Duero, 5, 1°, 28001, Madrid, Spain; 4 Hospital Sierrallana, Torrelavega, B°. de Ganzo, s/n 39300, Spain; 5 King Christian the Xth Hospital, Toldbodgade 3, 6300 Gråsten, Denmark; 6 Satakunta Central Hospital, Rauman aluesairaala, Steniuksenkatu 2, 26100 Rauma, Finland; 7 Hôpital Lapeyronie, 371, avenue du Doyen Gaston Giraud 34295 Montpellier Cedex 5, France; 8 Charité – University Medicine Berlin, Chariteplatz 1, 10117 Berlin, Germany; 9 Waterford Regional Hospital, Dunmore Road, Waterford, Co Waterford, Ireland; 10 Catholic University of Sacred Heart, Largo F. Vito, 1 – 00168, Rome, Italy; 11 Medisch Spectrum Twente, Haaksbergerstraat 55, 7513 ER, Enschede, The Netherlands; 12 Medical University of Lublin, al. Rac^3^awickie 1, 20-095, Lublin, Poland; 13 Szpital Wojewodzki im. Jana Biziela, Ul. Kornela Ujejskiego 65, 85-104 Bydgoszcz, Poland; 14 Hospital General de Castellón, Avenida Benicasim S/N 12004 Castellón, Spain; 15 Hudiksvall Medical Clinic, 824 81 Hudiksvall, Sweden; 16 Kings College Hospital, Strand, London WC2R 2LS UK; 17 Meram Medical Faculty, Selcuk University, Konya 42090, Turkey; 18 Rheumatology Institut, Srpskih Junaka 2, 18205 Niška Banja, Serbia; 19 Taylor Hospital, 175 East Chester Pike, Ridley Park, Pennsylvania, PA 19078USA; 20 Hospital San Juan Bautista, Avenida Illia 200, K4700ABO, Catamarca, Argentina; 21 New York University Hospital for Joint Diseases, 301 East 17th Street, New York, New York 10003, USA

## Abstract

**Introduction:**

We analyzed the prevalence of cardiovascular (CV) disease in patients with rheumatoid arthritis (RA) and its association with traditional CV risk factors, clinical features of RA, and the use of disease-modifying antirheumatic drugs (DMARDs) in a multinational cross-sectional cohort of nonselected consecutive outpatients with RA (The Questionnaires in Standard Monitoring of Patients with Rheumatoid Arthritis Program, or QUEST-RA) who were receiving regular clinical care.

**Methods:**

The study involved a clinical assessment by a rheumatologist and a self-report questionnaire by patients. The clinical assessment included a review of clinical features of RA and exposure to DMARDs over the course of RA. Comorbidities were recorded; CV morbidity included myocardial infarction, angina, coronary disease, coronary bypass surgery, and stroke. Traditional risk factors recorded were hypertension, hyperlipidemia, diabetes mellitus, smoking, physical inactivity, and body mass index. Unadjusted and adjusted hazard ratios (HRs) (95% confidence interval [CI]) for CV morbidity were calculated using Cox proportional hazard regression models.

**Results:**

Between January 2005 and October 2006, the QUEST-RA project included 4,363 patients from 48 sites in 15 countries; 78% were female, more than 90% were Caucasian, and the mean age was 57 years. The prevalence for lifetime CV events in the entire sample was 3.2% for myocardial infarction, 1.9% for stroke, and 9.3% for any CV event. The prevalence for CV risk factors was 32% for hypertension, 14% for hyperlipidemia, 8% for diabetes, 43% for ever-smoking, 73% for physical inactivity, and 18% for obesity. Traditional risk factors except obesity and physical inactivity were significantly associated with CV morbidity. There was an association between any CV event and age and male gender and between extra-articular disease and myocardial infarction. Prolonged exposure to methotrexate (HR 0.85; 95% CI 0.81 to 0.89), leflunomide (HR 0.59; 95% CI 0.43 to 0.79), sulfasalazine (HR 0.92; 95% CI 0.87 to 0.98), glucocorticoids (HR 0.95; 95% CI 0.92 to 0.98), and biologic agents (HR 0.42; 95% CI 0.21 to 0.81; *P *< 0.05) was associated with a reduction of the risk of CV morbidity; analyses were adjusted for traditional risk factors and countries.

**Conclusion:**

In conclusion, prolonged use of treatments such as methotrexate, sulfasalazine, leflunomide, glucocorticoids, and tumor necrosis factor-alpha blockers appears to be associated with a reduced risk of CV disease. In addition to traditional risk factors, extra-articular disease was associated with the occurrence of myocardial infarction in patients with RA.

## Introduction

Rheumatoid arthritis (RA) is associated with increased mortality, which is predominantly due to accelerated coronary artery and cerebrovascular atherosclerosis [1], a phenomenon that occurs in established and early RA [2-5]. Cardiovascular (CV) events occur approximately a decade earlier in RA than in the general population [6], suggesting that RA, similarly to diabetes mellitus, is an independent risk factor for premature ischemic heart disease [7,8].

The use of methotrexate is associated with a significantly lower risk for CV events in RA patients compared with patients who had never used disease-modifying antirheumatic drugs (DMARDs) [9]. Suissa and colleagues [10] found a negative association between the rate of myocardial infarction and the current use of any DMARD in a case control study. A study from Sweden [11] suggested that the risk for developing first CV events in RA was lower in patients who were treated with tumor necrosis factor-alpha (TNF-α) blockers. Our objective was to analyze the prevalence of CV morbidity in a large international sample of RA patients, its association with traditional CV risk factors, clinical features of RA, and with the use of DMARDs.

## Materials and methods

QUEST-RA is short for Questionnaires in Standard Monitoring of Patients with Rheumatoid Arthritis. It is an international effort to perform an identical cross-sectional review of 100 nonselected consecutive outpatients with RA in three or more rheumatology clinics in several countries [12]. Countries that joined QUEST-RA by June 2006 were Denmark, Finland, France, Germany, Ireland, Italy, the Netherlands, Poland, Serbia, Spain, Sweden, Turkey, the UK, the USA, and Argentina. Approval for the study was obtained from local internal review boards or ethics committees, and patients signed an informed consent form.

### Clinical evaluation

Patients were assessed according to a standard protocol to evaluate rheumatoid arthritis (SPERA) [13]. The rheumatologists performed a clinical assessment including swollen and tender joint counts. Information concerning extra-articular features and comorbidities, including CV events, was established by a record review, a detailed clinical examination, and asking the patient at the time of the visit. The use of all DMARDs, including dates of start and discontinuation of each DMARD, was recorded. The most recent rheumatoid factor (RF) values were collected; RF was considered positive or negative according to the local reference values any time over the course of the disease. No training as to how to collect data or to perform joint counts was provided, and the study was intended to reflect routine clinical practice. All patients had fulfilled the American College of Rheumatology 1987 criteria for the classification of RA during the course of the disease [14].

The presence of subcutaneous nodules, lung disease (nodules, fibrosis, or pleuritis), Felty syndrome, vasculitis, pericarditis, and scleritis was counted for extra-articular disease. CV events included myocardial infarction, angina, coronary disease, coronary bypass surgery, and stroke. Dates of these events were recorded. These data were based on participating rheumatologists' reports of CV events on their patients and no other confirmation was required. The presence of hyperlipidemia, diabetes mellitus, and hypertension was recorded. No type specifications for hyperlipidemia or diabetes mellitus were made.

### Patient self-report

The patients completed an expanded self-report health questionnaire that was first translated to each language; the study is explained elsewhere in more detail [12]. The questionnaire included the Health Assessment Questionnaire (HAQ) [15], years of education, height and weight for body mass index (BMI), and lifestyle choices such as smoking and frequency of physical exercise. Smokers were classified as 'nonsmokers' and 'ever-smokers', including 'current smokers'. Obesity was defined as BMI of greater than 30. 'Physical inactivity' included patient responses 'no exercise' or '1 to 2 times a month' versus exercises 'regular exercise one or more times a week'.

### Statistical analyses

Data are presented as means with standard deviations (SDs) and percentages with 95% confidence intervals (CIs). The Student *t *test and chi-square test were used for comparison between groups.

The prevalence of CV events was calculated including all patients in the cohort who reported to have had a CV event. Patients with a CV event before the diagnosis of RA were excluded from the risk factor analyses. Time-to-event was calculated from the date of diagnosis of RA; patients with no CV event were censored at the date of evaluation. Univariate Cox proportional hazard regression models were computed to estimate the risk of any CV event including each of the traditional CV risk factors and disease characteristics as independent variables in separate models. Second, multivariate Cox proportional hazard regression models were computed to estimate the risk of all CV events, myocardial infarction, and stroke. Variables that were included in these models were age, gender, presence of RF, extra-articular disease, hypertension, hyperlipidemia, diabetes, smoking, obesity, physical inactivity, and country. Multivariate analyses were performed including all patients who had a CV event after a diagnosis of RA and separately for 'high' and 'low' CV prevalence countries (that is, countries above and below the median, respectively).

Time of exposure to each DMARD was calculated as the time (in years) that elapsed between the date of start of a DMARD and the date of discontinuation, CV event, or date of evaluation, which ever happened first. Each DMARD was analyzed independently in a Cox regression model, first unadjusted and then adjusted for age, gender, disease activity/severity (DAS 28 [disease activity score using 28 joint counts] and HAQ), RA characteristics (RF positivity and presence of extra-articular manifestations), and the presence of traditional CV risk factors (hypertension, hyperlipidemia, diabetes, smoking, and obesity).

## Results

### Patients

Data collection began in January 2005, and in October 2006 the QUEST-RA project included 4,363 patients at 48 sites in 15 countries. Concerning demographic variables, this cohort represents a typical RA population: 78% of patients were female, 90% were Caucasian, the mean (SD) age was 57 (14) years, the mean disease duration was 11 (9) years, and the mean duration of education was 10 (4) years. Overall, 74% of patients had positive RF, ranging from 61% in Germany to 90% in Argentina. Extra-articular disease was present in 24% of patients, ranging from less than 15% in Italy and the Netherlands to 34% in Denmark (Table 1).

**Table 1:** Patient characteristics in the QUEST-RA study per country

Country	Number of sites	Number of patients	Age in years (mean)	Female (%)	Years of education (mean)	Disease duration in years (mean)	RF-positive (%)	Extra-articular disease^a ^(%)	Cardiovascular events (all)^b ^(%)
Denmark	3	301	58	76	11	12	74	34	9.97
Finland	3	304	59	72	10	13	75	18	11.18
France	4	389	55	78	11	13	75	21	3.60
Germany	3	225	59	84	10	13	61	36	17.78
Ireland	3	225	57	64	11	11	81	30	6.67
Italy	4	336	61	78	8	10	73	13	8.93
Netherlands	3	317	59	68	11	9	69	13	9.46
Poland	7	642	53	86	12	11	71	33	11.84
Spain	3	301	60	74	10	11	71	23	9.97
Sweden	3	248	59	72	10	12	82	23	8.47
UK	3	126	60	78	12	15	84	30	10.32
Turkey	3	309	52	85	7	11	69	18	6.80
Serbia	1	100	59	88	8	10	71	23	9.00
USA	3	294	57	72	14	9	70	24	11.56
Argentina	2	246	51	90	9	10	90	26	3.66
Total	48	4,363	57 ± 1	78	10 ± 4	11 ± 9	73.7	24.3	9.31

^a^Includes nodules, pulmonal fibrosis, pericarditis, vasculitis, Felty syndrome, and scleritis. ^b^Myocardial infarction, angina, coronary heart disease, coronary bypass surgery, or stroke. QUEST-RA, Questionnaires in Standard Monitoring of Patients with Rheumatoid Arthritis; RF, rheumatoid factor.

### Cardiovascular morbidity

The overall prevalence of CV morbidity (myocardial infarction, angina, coronary disease, or stroke) was 9.3%, with considerable variation between countries (less than 5% in Argentina and France and greater than 10% in Finland, Germany, Poland, the UK, and the USA) (Table 1). CV events were more prevalent in men than in women (Table 2). The overall prevalence for the whole cohort of lifetime myocardial infarction was 3.2%, and the prevalence for stroke was 1.9%.

**Table 2:** Cardiovascular morbidity and risk factors in the QUEST-RA study by gender

	Male		Female	
	Mean/%	95% CI	Mean/%	95% CI

Myocardial infarction^a^	8	6–10	2	1–2
Stroke^b^	3	2–4	2	1–2
Cardiovascular (all)^a,c^	16	14–18	7	7–8
Age in years^a^	58.9	58.1–59.7	56.3	55.8–56.7
Disease duration in years^a^	8.8	8.2–9.4	10.0	9.7–10.3
Rheumatoid factor-positive	74	71–76	72	70–73
Hypertension	31	28–34	32	31–34
Hyperlipidemia	13	11–16	14	13–15
Diabetes mellitus^b^	10	8–12	7	6–8
Smoking ever^a^	68	65–71	37	35–38
Smoking now^a^	26	23–29	15	14–17
Body mass index ≥30^a^	13	11–15	19	18–20
Physical inactivity^d^	68	65–71	73	72–75

^a^*P *< 0.001. ^b^*P *< 0.05. ^c^Myocardial infarct, angina, coronary heart disease, bypass, or stroke. ^d^*P *< 0.01. CI, confidence interval; QUEST-RA, Questionnaires in Standard Monitoring of Patients with Rheumatoid Arthritis.

### Traditional cardiovascular risk factors

The prevalence of CV risk factors was 33% for hypertension, 14% for hyperlipidemia, 8% for diabetes, 43% for smoking ever, 72% for physical inactivity, and 18% for obesity. Diabetes was more frequent in men (10%) than in women (7%) (Table 2). More men than women had ever smoked (68% versus 37%; *P *= 0.0001) or were current smokers (26% versus 15%; *P *= 0.0001).

In a univariate Cox regression analysis, extra-articular disease was statistically significantly associated with CV morbidity (Table 3). Among traditional CV risk factors, age, gender, hypertension, hyperlipidemia, ever-smoking, and diabetes showed a statistically significant association with CV events.

**Table 3:** Univariate analyses for cardiovascular morbidity in patients with rheumatoid arthritis in the QUEST-RA study

	Cardiovascular morbidity (percentage of patients)				
	No	Yes	Hazard ratio	95% CI	*P *value

Rheumatoid variables					
Rheumatoid factor	71.7	77.0	1.02	0.76–1.37	0.881
Extra-articular disease^a^	23.0	43.3	1.52	1.19–1.95	0.001
					
Traditional risk factors					
Age in years (mean)	55.8	66.4	1.05	1.04–1.06	0.000
Gender (female)	79.4	64.0	0.42	0.33–0.54	0.000
Hypertension	28.7	62.9	2.97	2.31–3.83	0.000
Hyperlipidemia	11.3	34.0	3.19	2.47–4.13	0.000
Diabetes	6.9	15.8	2.09	1.50–2.92	0.000
Smoking ever	42.6	51.2	1.60	1.25–2.04	0.000
Smoking now	18.3	11.8	0.80	0.55–1.18	0.260
Obesity	17.5	18.3	1.34	0.96–1.86	0.082
Physical inactivity	71.9	73.5	1.00	0.75–1.33	0.993

^a^Includes nodules, pulmonal fibrosis, pericarditis, vasculitis, Felty syndrome, and scleritis. CI, confidence interval; QUEST-RA, Questionnaires in Standard Monitoring of Patients with Rheumatoid Arthritis.

In a multivariate Cox regression analysis, older age, male gender, hypertension, hyperlipidemia, and ever-smoking were independently associated with occurrence of CV events (Table 4). Extra-articular RA (hazard ratio [HR] 2.26; 95% CI 1.29 to 3.97), hyperlipidemia (HR 3.51; 95% CI 1.98 to 6.21), and ever-smoking (HR 3.20; 95% CI 1.74 to 5.90) were all associated with myocardial infarction although association of extra-articular disease was not statistically significant in 'low prevalence' (of CV disease) countries (Table 4). Hypertension (HR 2.81; 95% CI 1.49 to 5.30) and diabetes (HR 2.23; 95% CI 1.12 to 4.44) were associated with stroke; associations were statistically significant for hypertension in 'high prevalence' countries and for diabetes in 'low prevalence' countries (Table 4).

**Table 4:** Multivariate model for cardiovascular morbidity in the QUEST-RA study

	Cardiovascular events (all)^a ^hazard ratio (95% CI)		
	All countries	Countries with high prevalence	Countries with low prevalence
Age	1.04 (1.03–1.06)^b^	1.05 (1.03–1.07)^b^	1.04 (1.01–1.06)^c^
Gender (female)	0.49 (0.36–0.66)^b^	0.45 (0.31–0.66)^b^	0.55 (0.32–0.94)^c^
Rheumatoid factor	0.95 (0.68–1.33)	0.95 (0.64–1.41)	0.90 (0.46–1.73)
Extra-articular disease	1.33 (1.00–1.78)	1.28 (0.92–1.80)	1.60 (0.91–2.84)
Hypertension	1.95 (1.44–2.63)^b^	1.85 (1.29–2.64)^c^	2.23 (1.28–3.90)^c^
Hyperlipidemia	2.41 (1.78–3.27)^b^	2.16 (1.50–3.10)^b^	3.24 (1.86–5.65)^b^
Diabetes	1.34 (0.92–1.96)	1.05 (0.65–1.69)	2.20 (1.18–4.09)^c^
Ever-smoking	1.56 (1.16–2.12)^c^	1.46 (1.01–2.11)^d^	1.88 (1.10–3.22)^c^
Obesity	1.16 (0.81–1.66)	1.52 (1.01–2.27)^d^	0.57 (0.25–1.28)
Physical inactivity	0.93 (0.67–1.30)	1.11 (0.75–1.65)	0.61 (0.33–1.10)
			
	Myocardial infarction hazard ratio (95% CI)		

	All countries	Countries with high prevalence	Countries with low prevalence

Age	1.05 (1.02–1.08)^c^	1.04 (1.01–1.08)^c^	1.06 (1.00–1.13)^c^
Gender (female)	0.46 (0.27–0.80)^c^	0.55 (0.29–1.06)	0.37 (0.13–1.04)
Rheumatoid factor	0.68 (0.36–1.31)	0.68 (0.31–1.48)	0.82 (0.23–2.91)
Extra-articular disease	2.26 (1.29–3.97)^c^	2.26 (1.17–4.37)^c^	2.13 (0.71–6.35)
Hypertension	1.45 (0.83–2.53)	1.10 (0.57–2.15)	2.19 (0.72–6.71)
Hyperlipidemia	3.51 (1.98–6.21)^b^	3.51 (1.80–6.84)^b^	4.34 (1.41–13.33)^c^
Diabetes	1.18 (0.59–2.38)	1.27 (0.56–2.90)	1.01 (0.24–4.29)
Ever-smoking	3.20 (1.74–5.90)^b^	2.47 (1.22–4.99)^c^	12.14 (2.50–59.05)^c^
Obesity	0.74 (0.34–1.62)	0.59 (0.22–1.57)	1.36 (0.37–5.01)
Physical inactivity	0.88 (0.46–1.65)	0.96 (0.45–2.07)	0.87 (0.24–3.12)
			
	Stroke hazard ratio (95% CI)		

	All countries	Countries with high prevalence	Countries with low prevalence

Age	1.02 (0.99–1.05)	1.03 (1.00–1.06)	0.99 (0.93–1.05)
Gender (female)	0.59 (0.31–1.12)	0.66 (0.31–1.42)	0.37 (0.10–1.39)
Rheumatoid factor	0.95 (0.45–2.00)	0.88 (0.38–2.06)	2.67 (0.40–17.71)
Extra-articular disease	1.41 (0.77–2.58)	1.43 (0.69–2.94)	1.31 (0.40–4.30)
Hypertension	2.81 (1.49–5.30)^c^	2.95 (1.43–6.11)^c^	2.37 (0.61–9.20)
Hyperlipidemia	1.18 (0.60–2.31)	0.69 (0.27–1.76)	3.69 (1.16–11.78)^c^
Diabetes	2.23 (1.12–4.44)^d^	1.70 (0.71–4.07)	4.63 (1.31–16.40)^c^
Ever-smoking	1.66 (0.89–3.09)	1.74 (0.82–3.69)	1.31 (0.37–4.63)
Obesity	0.72 (0.31–1.68)	0.77 (0.28–2.08)	0.56 (0.11–2.69)
Physical inactivity	1.01 (0.50–2.03)	1.30 (0.59–2.87)	0.52 (0.13–2.09)

High and low values of each cardiovascular event are based on the median. All variables are included simultaneously in the model, adjusted for country. ^a^Myocardial infarct, angina, coronary heart disease, bypass, or stroke. ^b^*P *< 0.001. ^c^*P *< 0.01. ^d^*P *< 0.05. CI, confidence interval; QUEST-RA, Questionnaires in Standard Monitoring of Patients with Rheumatoid Arthritis.

### Disease-modifying antirheumatic drugs and glucocorticoids

Patients who had hypertension were treated less frequently with methotrexate and biologic agents compared with patients who had no hypertension, but the former were treated more frequently with leflunomide and glucocorticoids (data not shown). Table 5 shows the HR for the occurrence of CV events by year of exposure to each DMARD, when adjusted to age, gender, disease activity, and traditional risk factors. One year of methotrexate use was associated with 15%, 18%, and 11% decreases of risk for all CV events, myocardial infarction, and stroke, respectively. Leflunomide was also associated with a reduced risk of CV events, and glucocorticoids and sulfasalazine were associated with a small but significantly reduced risk of all CV events. A lower risk for all CV events and myocardial infarction was also associated with a longer exposition-duration to TNF-α blockers (HR 0.42; 95% CI 0.21 to 0.81; *P *< 0.05).

**Table 5:** Years of exposure to disease-modifying antirheumatic drugs and cardiovascular morbidity in patients with rheumatoid arthritis in the QUEST-RA study

	HR^a ^(95% CI) CV all types	HR^b ^(95% CI) CV all types	HR^c ^(95% CI)		
			
			CV all types	Myocardial infarction	Stroke
Methotrexate	0.82 (0.79–0.86)^d^	0.84 (0.80–0.87)^d^	0.85 (0.81–0.89)^d^	0.82 (0.74–0.91)^d^	0.89 (0.82–0.98)^e^
Glucocorticoids	0.94 (0.92–0.97)^d^	0.95 (0.93–0.97)^d^	0.95 (0.92–0.98)^d^	0.96 (0.91–1.00)	0.98 (0.93–1.03)
Antimalarials	0.94 (0.91–0.98)^f^	0.95 (0.91–0.99)^f^	0.98 (0.94–1.02)	0.94 (0.85–1.03)	0.87 (0.76–1.01)
Sulfasalazine	0.91 (0.87–0.96)^d^	0.92 (0.88–0.97)^f^	0.92 (0.87–0.98)^f^	0.82 (0.69–0.98)^e^	0.90 (0.79–1.03)
Gold	0.96 (0.92–1.00)^e^	0.96 (0.92–1.00)^e^	0.99 (0.95–1.03)	1.04 (0.98–1.10)	0.98 (0.89–1.07)
Leflunomide	0.52 (0.38–0.72)^d^	0.55 (0.41–0.75)^d^	0.59 (0.43–0.79)^f^	0.52 (0.26–1.06)	0.91 (0.65–1.28)
TNF-α blockers	0.67 (0.53–0.85)^f^	0.71 (0.56–0.89)^f^	0.64 (0.49–0.83)^f^	0.42 (0.21–0.81)^e^	0.64 (0.39–1.05)

^a^Crude hazard ratio. ^b^Adjusted hazard ratio by age and gender. ^c^Adjusted hazard ratio by age, gender, disease activity/severity (DAS 28 [disease activity score using 28 joint counts] and Health Assessment Questionnaire), rheumatoid arthritis characteristics (rheumatoid factor-positive and extra-articular manifestations), and traditional cardiovascular risk factors (hypertension, hyperlipidemia, diabetes, smoking ever, and obesity). ^d^*P *< 0.001. ^e^*P *< 0.05. ^f^*P *< 0.01. CI, confidence interval; CV, cardiovascular; QUEST-RA, Questionnaires in Standard Monitoring of Patients with Rheumatoid Arthritis; TNF-α, tumor necrosis factor-alpha.

## Discussion

This study provides further support to the concept that a prolonged use of DMARDs, glucocorticoids, and TNF-α blockers is associated with a reduced risk of CV events. Furthermore, extra-articular RA was found to be associated with the occurrence of myocardial infarction, and the role of traditional risk factors for CV morbidity was confirmed.

### Incidence and prevalence of cardiovascular disease in rheumatoid arthritis

Patients with RA are 30% to 60% more likely to suffer a CV event compared with the general population [16,17], especially myocardial infarction [18-20], whereas the incidence and prevalence of stroke generally have been reported to be similar in RA as in the general population or in patients with osteoarthritis [18,20]. Only one study found higher prevalence of stroke in RA than in controls [16]. We found a lower prevalence of stroke compared with other cross-sectional studies [16,18], although comparative data for the reference from general populations were not available in our study.

The crude prevalence of CV events differed between countries (Table 1). Results in the present study, to some extent, are similar to those in the World Health Organization MONICA (Multinational Monitoring of Trends and Determinants in Cardiovascular Disease) project, in which the highest rates of myocardial infarction were seen in Finland, Poland, and the UK and lowest in the Mediterranean countries [21], possibly related to the Mediterranean diet and lifestyle, which are associated with a greater than 50% decrease of all-cause and cause-specific mortality in the general population [22]. However, our data are not directly comparable to the MONICA study, in which participants were less than 65 years old, whereas the QUEST-RA patients are predominantly older females.

### Traditional risk factors for cardiovascular disease

In our study, the frequency of CV events was double in men compared with women (four times for myocardial infarction) and does not provide any surprises compared with observations in the general population [21]. In univariate analyses, all traditional CV risk factors, except obesity and physical inactivity, were associated with CV morbidity, and in multivariate models, hypertension, hyperlipidemia, diabetes, and ever-smoking remained independent risk factors. Thus, our results confirm the role of traditional risk factors concerning CV morbidity in patients with RA. However, it needs to be recognized that our analyses are restricted to people who survived following a CV event due to the cross-sectional nature of the study and some of the predictors being identified may be predictors of survival following a CV event rather than the occurrence of the event. Furthermore, we did not collect data on family history of CV disease, which appears a significant risk factor for CV morbidity also in patients with RA [23].

Physical inactivity is common in the general population and a frequent consequence of arthritis. In the general population, the frequency of weekly physical activity of three or more times is associated with reduced CV morbidity [24]. In the present study, only 13% of patients exercised three or more times a week [25]. Physical inactivity (exercise frequency '1 to 2 times a month' and 'none') was not associated with CV disease in our analyses.

### Cardiovascular morbidity and rheumatoid arthritis-related risk factors

RA is associated with premature and accelerated atherosclerosis [16,26]. Carotid artery ultrasound and coronary angiography have shown that atheromatosis is more frequent in RA than controls [27,28] and is related to the extent of radiographic damage of joints, which, in turn, is indicative of sustained inflammation [29]. Moreover, multivessel coronary artery calcifications in RA are related to smoking and an elevated sedimentation rate [4]. An elevated C-reactive protein at baseline predicted CV mortality over the follow-up of 10 years in patients with inflammatory polyarthritis in general and particularly in RF-positive patients [30,31]. Some processes intrinsic to the pathogenesis of RA play important roles in CV damage and its clinical consequences: abnormal endothelial function [32], autoantibodies against oxidized low-density lipoprotein [33], and levels of serum mannose-binding leptin [34].

Van Halm and colleagues [9] found an association between CV disease and seropositive and erosive RA, and Turesson and colleagues [8,35] between CV morbidity and mortality and extra-articular disease. The present study lends further support to the significant association between extra-articular disease and CV morbidity, and this observation underlines higher CV morbidity in severe RA.

### Antirheumatic drugs and cardiovascular disease

Some of the medications used to treat RA might increase the risk for CV morbidity. The risk of myocardial infarction is increased in nonsteroidal anti-inflammatory drug (NSAID) and cyclooxygenase 2 inhibitor users, especially with rofecoxib [36,37].

Glucocorticoids might increase CV morbidity by promoting proatherosclerotic lipid profiles [38,39]. Two recent articles show an increased rate of CV events with long-term use of glucocorticoids [10,40]. However, Davis and colleagues [41] found no association between cumulative glucocorticoid exposure and CV events in RA patients followed for a median of 15 years, after adjusting for other CV risk factors and markers of RA activity. In the present study, long-term use of glucocorticoids was independently associated with a reduced risk of 'all' CV events (Table 5).

The use of methotrexate has been associated with a significantly lower risk for CV events in RA patients compared with patients who had never used DMARDs [9]. Long-term follow-up of RA patients has shown that the use of methotrexate is significantly associated with reduced overall and CV mortality [42].

Suissa and colleagues [10], in a case control study, found that the use of DMARDs was associated with a decreased rate of myocardial infarction. However, Solomon and colleagues [40], in a case control study, found a higher risk for CV events in patients who had received cyclosporine, azathioprine, or leflunomide. In our study, longer use of DMARDs such as methotrexate, leflunomide, and sulfasalazine was associated with a decreased risk of CV disease. The association remained statistically significant for myocardial infarction with methotrexate (*P *< 0.001) and sulfasalazine (*P *< 0.05), even when adjusted for disease severity and traditional risk factors such as smoking, which is a risk factor for both CV disease and RA [43]. In contrast, the use of antimalarials and intramuscular gold was not associated with a change of the risk of CV disease. In one report, atherogenic lipid profiles of RA patients improved after specific therapy for arthritis, primarily due to the increase of serum high-density lipoprotein cholesterol levels [44].

With respect to TNF-α blockers, infliximab may improve endothelial function in RA after 12 weeks of therapy [45], suggesting that inflammation is a mediator of endothelial dysfunction, although such beneficial effects do not appear to sustain for a long time [46,47]. A recent study suggested that the risk for developing first CV events in RA is lower in patients treated with TNF-α blockers [11]. However, that study did not control for most of the traditional risk factors for accelerated atherosclerosis. On the other hand, two case control studies showed no reduction of infarction rate in RA with TNF-α blockers [10,41]. Other recent reports from large databases show discordant results on CV disease incidence in TNF users versus nonusers [48-50]. In the present study, longer use of TNF-α blockers was associated with a reduced risk of CV disease although limited availability of biologics might interfere with the results. Furthermore, patients with suspected CV disease may not be prescribed biologic agents.

Several limitations are to be recognized. First, QUEST-RA is a cross-sectional study with a possible left censorship concerning CV events. Many patients may have experienced a fatal CV event and, therefore, could not be included in this study. Second, all data are based on participating rheumatologists' reports and no verification of data was performed. Therefore, it is possible that some data could be missed. Third, data on the longitudinal use of NSAIDs or cyclooxygenase 2 inhibitors were not collected. Fourth, the extent of radiographic erosions (representing cumulative disease activity) was not analyzed in the present study. Despite limitations, QUEST-RA is a unique program that has succeeded to date in collecting data on clinical RA patients according to an identical protocol in various locales in various countries and various cultures and provides data that are not available from any other resources at this time.

## Conclusion

Our study provides further support of the influence of both traditional and RA-specific risk factors in the development of CV events, especially myocardial infarction. As assessed by this study, the risk was lower with the prolonged use of methotrexate, sulfasalazine, glucocorticoids, leflunomide, and TNF-α blockers.

## Abbreviations

BMI = body mass index; CI = confidence interval; CV = cardiovascular; DMARD = disease-modifying antirheumatic drug; HAQ = Health Assessment Questionnaire; HR = hazard ratio; MONICA = Multinational Monitoring of Trends and Determinants in Cardiovascular Disease; NSAID = nonsteroidal anti-inflammatory drug; QUEST-RA = Questionnaires in Standard Monitoring of Patients with Rheumatoid Arthritis; RA = rheumatoid arthritis; RF = rheumatoid factor; SD = standard deviation; TNF-α = tumor necrosis factor-alpha.

## Competing interests

The authors declare that they have no competing interests.

## Authors' contributions

The QUEST-RA study was designed and conducted by TS and TP. All authors participated in data collection concerning their clinical patients. Analyses for the present report were designed and coordinated by AN and performed by MAD. AN drafted the manuscript with the help of TS. All authors read and approved the final manuscript.
